# Hippocampal and diencephalic pathology in developmental amnesia

**DOI:** 10.1016/j.cortex.2016.09.016

**Published:** 2017-01

**Authors:** Anna M. Dzieciol, Jocelyne Bachevalier, Kadharbatcha S. Saleem, David G. Gadian, Richard Saunders, W.K. Kling Chong, Tina Banks, Mortimer Mishkin, Faraneh Vargha-Khadem

**Affiliations:** a University College London Great Ormond Street Institute of Child Health, London, UK; b Yerkes National Primate Research Center, Emory University, Atlanta, GA, USA; c National Institutes of Health, Bethesda, MD, USA; d Department of Radiology, Great Ormond Street Hospital for Children, London, UK

**Keywords:** Memory, Hypoxia-ischaemia, Hippocampus, Thalamus, Mammillary bodies, DA, developmental amnesia, IQ, intelligence quotient, MQ, memory quotient, CMS, Children's Memory Scale, WMS-III, Wechsler's Memory Scale, 3rd ed., AMT, anterior-mid thalamus, PT, posterior thalamus, Th-Auto, automatically segmented thalamus, MBs, mammillary bodies

## Abstract

Developmental amnesia (DA) is a selective episodic memory disorder associated with hypoxia-induced bilateral hippocampal atrophy of early onset. Despite the systemic impact of hypoxia-ischaemia, the resulting brain damage was previously reported to be largely limited to the hippocampus. However, the thalamus and the mammillary bodies are parts of the hippocampal-diencephalic network and are therefore also at risk of injury following hypoxic-ischaemic events. Here, we report a neuroimaging investigation of diencephalic damage in a group of 18 patients with DA (age range 11–35 years), and an equal number of controls. Importantly, we uncovered a marked degree of atrophy in the mammillary bodies in two thirds of our patients. In addition, as a group, patients had mildly reduced thalamic volumes. The size of the anterior-mid thalamic (AMT) segment was correlated with patients' visual memory performance. Thus, in addition to the hippocampus, the diencephalic structures also appear to play a role in the patients' memory deficit.

## 1 Introduction

Situated within the medial-temporal lobe, the hippocampus is vulnerable to damage during hypoxic-ischaemic episodes due to its high oxygen requirement and susceptibility to glutamate-induced neurotoxicity (Schmidt-Kastner & Freund, 1991). However, these systemic events are associated with a cascade of direct and secondary neuropathology (Faro & Windle, 1969) affecting multiple brain regions (Bachevalier and Meunier, 1996, Caine and Watson, 2000, Spiers et al., 2001). Thus, damage to regions outside of the hippocampus could either independently or secondarily contribute to the patients' memory deficits.

Hypoxic-ischaemic events are known to target directly diencephalic structures, including the thalamus (Faro and Windle, 1969, Kuwert et al., 1993, Low, 2004, Markowitsch et al., 1997, Sie et al., 2000, Thayyil et al., 2010) and the mammillary bodies (Johkura and Naito, 2008, Kumar et al., 2008, Vortmeyer et al., 1993). Caine and Watson's (2000) survey of neuropathological studies revealed that the thalamus was affected in 56% of anoxic patients. In addition to these direct effects, the mammillary bodies and nuclei of the thalamus can become damaged after injury to the hippocampus by anterograde degeneration (Bachevalier & Meunier, 1996). In a neuronal circuit, an injury can propagate following the path of anatomical connections. The hippocampus projects directly to the anterior thalamic nuclei and the mammillary bodies. An additional indirect connection relays the hippocampal signals from the mammillary bodies to the anterior thalamic nuclei through the mammillo-thalamic tract (Aggleton et al., 2005, Papez, 1937, Saunders et al., 2005). Indeed, hippocampal injury is known to produce degeneration of both the mammillary bodies (Loftus et al., 2000, Schubert and Friede, 1979) and the thalamus (Kodama et al., 2003).

The strong connectivity between the medial-temporal lobe and diencephalon is also reflected in functional changes. Patients with injury to the hippocampus show hypometabolism in the thalamus (Reed et al., 1999), while those with damage in the diencephalon have deficits in glucose metabolism (Reed et al., 2003) and reduced BOLD activation (Caulo et al., 2005) in the medial-temporal lobe. In the rodent, regions disrupted by lesions of the mammillothalamic tract include the hippocampus and the prefrontal and retrosplenial cortices (Vann & Albasser, 2009). The same networks are affected following damage to the hippocampus, suggesting that the functional effects of hippocampal and diencephalic damage cannot be fully distinguished. Although diencephalic damage has not been consistently identified in patients who had sustained episodes of hypoxia-ischaemia (Di Paola et al., 2008, Gold and Squire, 2006, Rempel-Clower et al., 1996), the few studies that have been reported are limited by small sample sizes, reducing sensitivity to the detection of such damage.

Here we describe a series of individuals who presented to the Department of Neuropsychology at Great Ormond Street Hospital for Children because of severe memory problems affecting their everyday life (Adlam et al., 2009, Gadian et al., 2000, Vargha-Khadem et al., 1997). These children and adolescents had impairments in episodic memory, with relative sparing of semantic memory (Vargha-Khadem et al., 1997). This disorder, labelled developmental amnesia (DA) is marked by extensive, bilateral damage to the hippocampus, resulting from early life exposure to hypoxic-ischaemic events (Cooper et al., 2015). Specifically, the criteria for this diagnosis are: (a) a verified episode of hypoxia-ischaemia *without* associated motor or global cognitive impairment regardless of precipitating aetiological factors; (b) visible reduction of hippocampal volumes bilaterally on MRI accompanied by quantified volume reduction above 25% relative to normal, and (c) severely impaired episodic memory. We have not seen to date any patient with *selective* episodic amnesia who does not show severe bilateral hippocampal volume reduction without a documented history of hypoxic-ischaemic encephalopathy in early life.

However, abnormalities in the thalamus have been previously found in patients with DA using voxel-based morphometry (Gadian et al., 2000, Vargha-Khadem et al., 2003). In addition, a high resolution imaging study of one case – HC (DA-6 in Adlam, Vargha-Khadem, Mishkin, & de Haan, 2005; Adlam et al., 2009; also included in the current series) revealed an absence of the mammillary bodies (Olsen et al., 2013). The present study aimed to (i) determine the incidence of diencephalic damage in a large group of patients with DA using manual and automatic segmentation techniques, and (ii) explore the role of diencephalic damage in the DA patients' memory disorder.

## 2 Materials and methods

### 2.1 Participants

We measured thalamic and mammillary body volumes in 18 patients with DA (mean age 20.3 years, range 11–35, 7 female) and an equal number of gender-matched controls of comparable age (mean 18.8 years, range 10–35, 7 female). There were no statistically significant differences in age between groups (*t*_34_ = −.60 ns).

The patients had presented with complaints of frequent memory problems that interfered with their everyday activities. These deficits prevailed despite relatively preserved intelligence, language abilities, and academic attainments. In all but one of our cases, the patients with DA attended mainstream schools, although, on transition from primary to secondary school, the majority required some degree of educational support to help them with organisational skills and with preparation for exams. Most patients left education by the age of 16 and did not progress either to a university or to vocational training. As adults, the patients had difficulty living independently and securing regular employment with prospects for advancement.

**Fig. 1:**
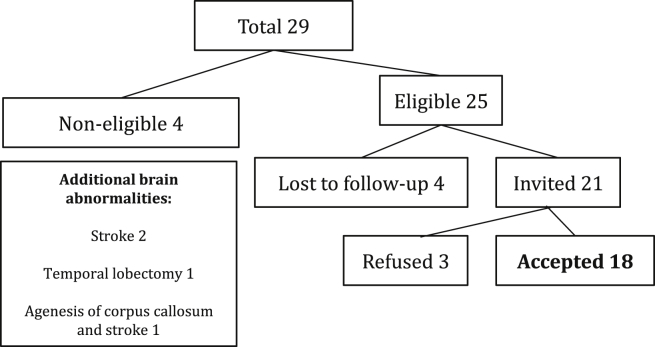
Recruitment of patients.

Subsequent examination revealed that these patients' memory problems were associated with bilateral loss of between 28% and 62% of hippocampal volume, compared to the mean hippocampal volume of an independent group of 65 healthy individuals. Neuroradiological assessment carried out by one of the authors (WKC) revealed additional damage to the fornix and the mammillary bodies, accompanied in some cases by abnormalities in the white matter and the cerebellum (Table 1). This damage was linked in each patient to episodes of hypoxia-ischaemia in the perinatal period or later in childhood (Table 2). While the aetiologies leading to hypoxia-ischaemia were varied, the most common were complications associated with premature birth (7 cases) or an acute adverse perinatal event (6 cases). Patients with evidence of additional brain abnormality (e.g., agenesis of the corpus callosum, stroke, temporal lobectomy) were excluded from the study (Fig. 1).

The controls, who were native speakers of English without any neurological or neuropsychological impairment, were recruited through advertisements at University College London, other local schools and colleges, and from a pool of healthy siblings of patients at Great Ormond Street Hospital.

### 2.2 Neuropsychology

Age-appropriate standardised tests of intelligence and memory were administered. Full-scale Intelligence Quotients (IQ) were calculated using the Wechsler Adult Intelligence Scale, 3rd ed. (WAIS-III) (Wechsler, 1998) or the Wechsler Intelligence Scale for Children, 4th ed. (WISC-IV) (Wechsler, 2003). This test contains four indices: Verbal Comprehension Index (VCI) measuring understanding of verbal concepts; Perceptual Reasoning Index (PRI) reflecting non-verbal perception and manipulation; Working Memory Index (WMI); and Processing Speed Index (PSI), measuring speed of non-verbal reasoning for routine visuo-motor tasks. General Memory Quotients (MQ) were measured using the Children's Memory Scale (CMS) (Cohen, 1997) or, for adults, the Wechsler's Memory Scale, 3rd ed. (WMS-III) (Wechsler, 1997), both of which provide indices of immediate and delayed memory for verbal and visual information.

Delayed Verbal Memory indices from the CMS and the WMS are both based on learning of paired-associated words, and recall of prose passages. However, Delayed Visual Memory scores are a composite of two subtests that differ according to the version of the test administered. In CMS, this score is composed of a Faces task (yes/no face recognition) and a Dot Location task (spatial recall involving choosing the location of previously presented checker pieces on a grid). In WMS-III, the Delayed Visual Memory score is composed of the Faces and Family Pictures subtests. Whereas the Faces task is similar to the one in the CMS (differing only in list length), the Family Pictures subtest is entirely different, requiring the participant to describe a previously presented picture.

The delayed Visual Memory index measures long-term non-verbal declarative memory performance, with stimuli designed to make verbal encoding difficult. While issues have been raised about the visual memory construct in healthy populations (Millis, Malina, Bowers, & Ricker, 1999), performance on this test is predictive of neurological damage in patients. For example, scores on WMS visual delayed index discriminate between temporal lobe epilepsy patients with right-sided lesions and those with left-sided lesions (Chiaravalloti et al., 2004, Doss et al., 2004).

Literacy and numeracy skills were assessed using the Wechsler Individual Achievement Test, 2nd ed. (WIAT-II) (Wechsler, 2001). Selected subtests were administered, including: Word Reading, Reading Comprehension, and Spelling, which were averaged into a measure of literacy; and Numerical Operations and Mathematical Reasoning, averaged to provide a combined measure of numeracy. Episodic memory was assessed with the Rivermead Behavioural Memory Test, 2nd ed. (RBMT) (Wilson, Cockburn, & Baddeley, 1985) for participants aged 11–35 years, and with the children's version for participants aged 8–10 years. This test measures patients' memory for everyday events with tasks including recall of names, stories, and routes, as well as prospective memory for actions. Semantic memory was measured using tests of vocabulary: Pyramids and Palm Trees Test (P&P) (Howard & Patterson, 1992) and British Picture Vocabulary Scale, 2nd ed. (BPVS-II) (Dunn, Whetton, & Pintilie, 1982). In the P&P Test, participants are presented with a target picture, and their task is to select its semantic associate out of two options (e.g., a pyramid is associated with a palm tree and not a pine tree). In BPVS-II, the participants hear a word (e.g., ‘arctic’ or ‘terpsichorean’), and are required to select a picture representing its meaning out of four options. Recall and recognition were measured using the Doors and People Test (D&P) (Baddeley, Emslie, & Nimmo-Smith, 1994), which consists of four equally challenging subtests, two assessing recognition and two assessing recall, and, within each of these pairs, one assessing visual ability and the other, verbal ability. To obtain *z*-scores of recall and recognition, performance was averaged across visual and verbal material.

### 2.3 MRI data acquisition

T1-weighted three dimensional Fast Low Angle Shot images were obtained using a 1.5 T Magnetom Avanto scanner (Siemens Healthcare, Erlangen, Germany). 176 contiguous sagittal slices were acquired with a field of view of 224 × 256 mm, and a voxel resolution of 1 × 1 × 1 mm. Echo time was 4.9 msec, repetition time was 11 msec, and flip angle was 15°. The acquisition time was approximately 5 min.

### 2.4 Structure segmentation

All manual measurements were performed twice by the same rater blind to subject identity. The average volume was used for further analysis.

#### 2.4.1 Hippocampus

Volumetric measurements of the hippocampus were carried out by one of the authors (DGG) (Gadian et al., 2000, Patai et al., 2015) using MEDx 3.43 (Medical Numerics, Inc., MD, USA). Detailed information on the procedures that were used for pre-processing of images, including identifying and outlining the boundaries of the hippocampus, is contained in Cooper et al. (2015). Briefly, the hippocampus was defined as a composite of CA1-4, dentate gyrus, subiculum, presubiculum, amygdalo–hippocampal transition area, and uncus, and was outlined in coronal sections.

#### 2.4.2 Thalamus

**Fig. 2:**
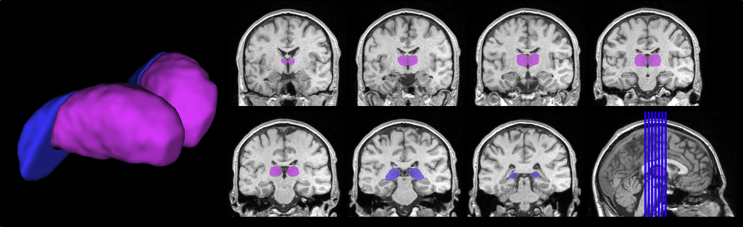
**Manually-outlined thalamic mask in a control participant**. AMT segment shown in purple, PT segment shown in blue.

Manual measurements of the anterior-mid and posterior thalamic (PT) segments and mammillary bodies were performed by one of the authors (AMD) using BRAINS2 (Magnotta et al., 2002). For the measurement of the thalamic volumes, images were re-formatted into 1-mm-thick coronal sections oriented perpendicular to the anterior commissure/posterior commissure line, and were corrected for head tilt. The thalamus was first outlined in every third section in the axial plane, as far ventrally as the posterior commissure. Then, independently of the axial outlines, the thalamus was segmented on every third section of the sagittal plane, from the midline to the level of the lateral pulvinar. The outlines in the axial and sagittal planes guided segmentation in the coronal sections, using 3D visualisation options available in BRAINS2 (Copenhaver et al., 2006). The coronal outlines were divided into anterior-mid thalamic (AMT) and posterior thalamic (PT) segments, with the most anterior outline of the PT placed at the last (most caudal) section through the habenula (Fig. 2). In some participants, the position of this section differed between the hemispheres. The AMT contained predominantly the anterior, medial dorsal, ventral anterior, ventral lateral, and the centromedian thalamic nuclei, and some rostroventral pulvinar. The PT contained most of the pulvinar (including medial, lateral, and inferior pulvinar nuclei), the caudal part of medial dorsal nucleus, the lateral and medial geniculate nuclei, and the reticular nucleus. These segments were chosen to enable reproducible tracing across brains.

**Fig. 3:**
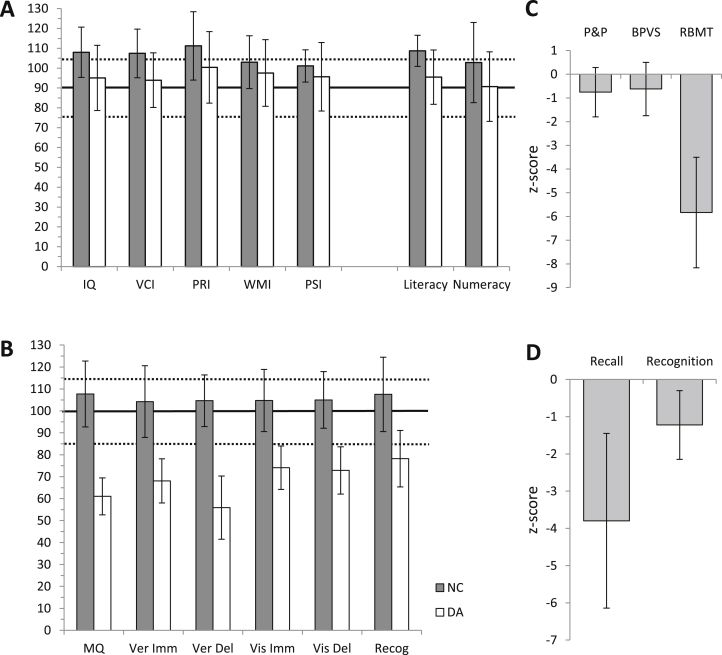
**Neuropsychology scores of patients with DA compared to those of the controls**. Solid and dotted horizontal lines show normative score and normative average range, respectively. A. Intelligence scores. B. Memory scores as measured by CMS and WMS-III. C. Semantic memory. There were no differences in semantic memory ability between patients and controls. D. Recall and recognition, measured using Doors and People Test.

In the coronal plane, the anterior limit of the AMT was placed where thalamic grey matter appeared medial to the internal capsule; the ventral boundary was placed at the dorsal limit of the hypothalamus and subthalamic nucleus; and the medial boundary was set at the lateral and third ventricles, the habenular nucleus and commissure, and the medial geniculate nucleus. The interthalamic adhesion (or massa intermedia), when present, was divided in half between the unilateral AMT segments. The ventrolateral boundary of the AMT was set to exclude the internal capsule and the lateral geniculate nucleus. However, due to diminishing grey matter/white matter contrast in the structural images, the PT outlines included the lateral geniculate nucleus and the white matter medial to the caudate nucleus and the hippocampus, causing the thalamic boundary to expand ventrally and laterally at the point of transition to the PT (see Fig. 2). The volumes of the AMT and PT were calculated by summing the cross-sectional areas measured in the coronal plane (Fig. 3).

In addition to the manual segmentation, the thalamus was also segmented automatically using the standard parameters in FSL-FIRST (FSL Integrated Registration and Segmentation Toolbox; Patenaude, Smith, Kennedy, & Jenkinson, 2011). Automatic segmentations were inspected for accuracy and minor errors were corrected manually by AMD, who remained blind to subject identity.

#### 2.4.3 Mammillary bodies

The mammillary bodies were classified as absent on the MRI image if no circular structures were visible at the ventral surface of the hypothalamus. If present, mammillary bodies were segmented by one of the authors (AMD) in every coronal section in the native space according to a protocol by Copenhaver et al. (2006). The protocol was modified to allow guide outlines in sagittal and axial planes to be placed in all sections intersecting the mammillary bodies. As with the thalamus, volumes were calculated by summing the areas of outlines in the coronal plane.

The volumetric measures of the mammillary bodies were in agreement with an independent neuroradiological assessment (see Table 1). With one exception (case DA-20), MB classified as abnormally small by the neuroradiologist were not suitable for volumetric measurement, and hence categorised as absent. Case DA-20's MBs had the lowest volume of all those that were segmented manually.

#### 2.4.4 Intracranial volume correction

Structure volumes were corrected for brain size by dividing them by the intracranial volumes (ICVs) obtained from the new segmentation procedure implemented in SPM8 (Statistical Parametric Mapping; http://www.fil.ion.ucl.ac.uk/spm/software/smp8/). All structure volumes are presented here as percentages of an individual's ICV.

## 3 Results

### 3.1 Neuropsychology

The patients' IQs were on average one standard deviation below that of the controls (*t*_33_ = 2.62, *p* = .013), though not significantly below that of the normative mean (*t*_16_ = −1.24 ns). The patients' Verbal Comprehension Index scores were also below those of the controls (*t*_34_ = −3.1, *p* = .004), however they did not differ from the normative mean (*t*_17_ = −1.87 ns). There were no statistically significant group differences in other IQ indices (PRI, *t*_34_ = −1.8 ns; WMI *t*_34_ = −1.1 ns; PSI, *t*_33_ = 1.2 ns). Patients' numeracy scores did not differ from the controls' (*F*_1,32_ = .2 ns, IQ-adjusted), although their literacy scores were reduced (*F*_1,32_ = 5.9, *p* = .021, IQ-adjusted). As previously, patients' literacy scores did not differ from the normative mean (*t*_17_ = −1.40 ns). On all memory indices, however, patients performed below both the control means and the normative means (adjusting for IQ: MQ, *F*_1,31_ = 93.0, *p* < .001; Visual Immediate, *F*_1,31_ = 36.4, *p* < .001; Visual Delayed, *F*_1,31_ = 54.1, *p* < .001; Verbal Immediate, *F*_1,31_ = 38.6, *p* < .001; Verbal Delayed, *F*_1,31_ = 108.2, *p* < .001; Recognition, *F*_1,31_ = 18.9, *p* < .001).

In contrast to the patients' severely impaired episodic memory, assessed with Rivermead Behavioural Memory Test (RBMT; *F*_1,17_ = 34.6, *p* < .001, IQ-adjusted), their semantic knowledge, measured by the P&P Test and the BPVS, was normal (adjusting for IQ: P&P, *F*_1,23_ = 1.2 ns; BPVS, *F*_1,23_ = .6 ns). Finally, while both their recall (*F*_1,26_ = 27.6, *p* < .001, IQ-adjusted) and recognition z-scores (*F*_1,26_ = 6.6, *p* = .016, IQ-adjusted) measured with the D&P Test were reduced compared to those of the controls, their recall z-score was significantly below their recognition z-score (*t*_12_ = −4.4, *p* < .001). Thus, whereas the patients' recall performance was, on average, 3.8 standard deviations below the control mean, their recognition performance was only 1.2 standard deviations below that of the controls (see Adlam et al., 2009).

### 3.2 Mammillary bodies

**Fig. 4:**
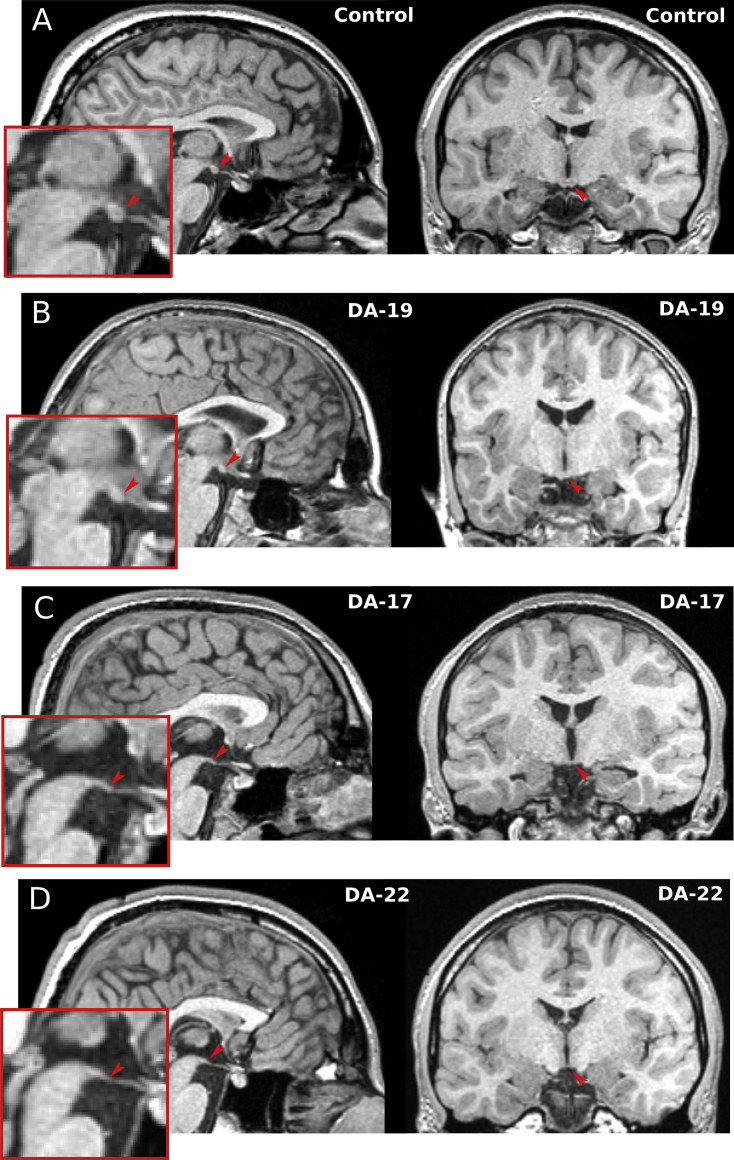
**Mammillary bodies in a control participant (A) and in three patients with DA (B–D)**. Arrowheads in midsagittal and coronal sections point to position of the MBs. **B**. Patient with MB volume within the control range. **C**–**D**. Two patients with MBs classified as absent.

**Fig. 5:**
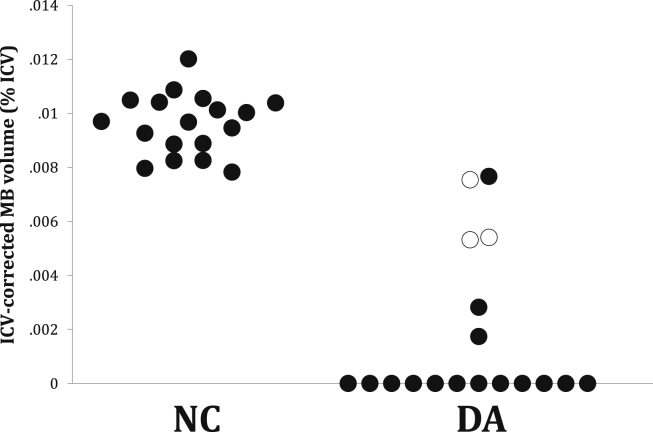
**Mammillary body volumes corrected for intracranial volumes (ICVs) in normal controls (NC) and in patients with DA**. Unfilled DA circles represent three patients who had sustained injury during childhood (between 4 and 15 years of age); filled DA circles, patients who had sustained injury neonatally.

The MBs were visible in the MRI images of all 18 control participants but only in six of the 18 patients (Fig. 4, Fig. 5). The MB volumes of these six were reduced compared to those of an equal number of matched controls (*F*_1,9_ = 18.97, *p* = .002, covaried for age). The hippocampal volumes of the 12 patients with no visible MBs were significantly lower than those of the six patients with visible MBs (*t*_16_ = −2.97, *p* = .009). There were no statistically significant differences between the subgroups on memory measures. Three of the 6 cases with visible MBs had suffered hypoxic-ischaemic injuries during childhood (between 4 and 15 years of age), whereas all 12 cases with no visible MB had sustained their hypoxia-ischaemia perinatally (Fig. 5).

### 3.3 Thalamic volumes

**Fig. 6:**
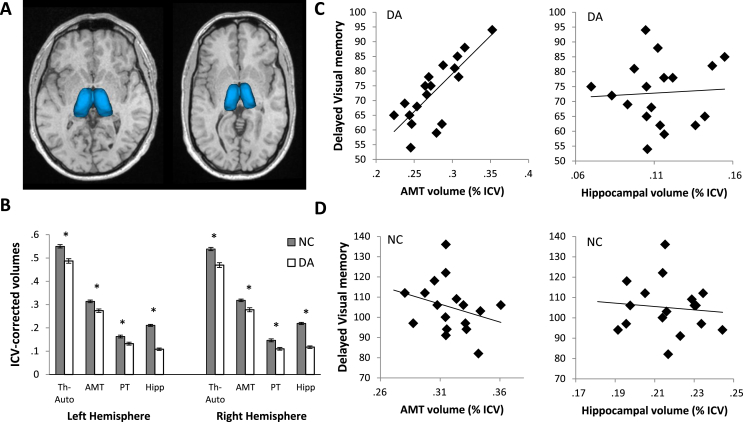
**Thalamus**. **A**. Automatically measured thalamic volumes in a control participant and in DA-1. The thalamic volume of the control case approximates the group median, whereas DA-1's volume is the lowest in its group. Images are not to scale. **B**. The DA patients had significantly lower volumes of AMT, PT, and the automatic thalamic measurement (Th-Auto) compared to those of the controls. **C**. There was a statistically significant correlation between the patients' scores on Delayed Visual Memory and their mean AMT volumes ICV-corrected for intracranial volumes (ICVs; *r*_17_ = .78, *p* < .001), but not with their hippocampal volumes (*r*_17_ = .06 ns). **D**. In the controls, there were no correlations between Delayed Visual Memory and either AMT volumes (*r*_16_ = −.28 ns) or volumes of the hippocampus (*r*_16_ = −.10 ns).

The patients with DA had reduced mean volumes of the automatically-segmented thalamus (Th-Auto; *F*_1,33_ = 33.3, *p* < .001; average 11% decrease), as well as manually-measured AMT (*F*_1,33_ = 18.3, *p* < .001; 12% decrease) and PT segments (*F*_1,33_ = 16.4, *p* < .001; 20% decrease), compared to matched controls and corrected for age at MRI scan (Fig. 6). These volume reductions were bilateral (left Th-Auto *F*_1,33_ = 28.4, *p* < .001; right Th-Auto *F*_1,33_ = 34.6, *p* < .001; left AMT *F*_1,33_ = 17.6, *p* < .001; right AMT *F*_1,33_ = 16.1, *p* < .001; left PT *F*_1,33_ = 11.6, *p* = .002; right PT *F*_1,33_ = 17.2, *p* < .001). In patients with DA, there were no correlations between the volumes of the mean, ICV-corrected thalamic segments and the volume of the hippocampus [Th-Auto *r_17_* = .23 ns; AMT *r_17_* = .19 ns; PT *r_17_* = .31 ns]. Mean ICV-corrected volumes of Th-Auto were correlated with volumes of AMT [*r_17_* = .80, *p* < .001], but not with PT [*r_17_* = −.01 ns]. Also, there were no correlations between volumes of AMT and PT [*r_17_* = .13 ns].

### 3.4 Correlations with indices of memory

A statistically significant correlation was found between the patients' AMT volumes and their scores on delayed visual memory (*r*_17_ = .78, *p* < .001; Fig. 5). This correlation remained statistically significant when only those patients who completed the CMS were included (6 cases received the CMS in the current study, and past CMS data were available for 4 additional cases who presently exceed the upper age range for CMS, *r*_9_ = .86, *p* = .001). After correcting for multiple comparisons, AMT volumes did not correlate with scores on other CMS/WMS subtests, nor with performance on RBMT and D&P tests, although there was a trend towards a correlation with General Memory (*r*_17_ = .44, *p* = .069). Likewise, PT volumes did not correlate with neuropsychological test scores.

Interestingly, there were no correlations between memory scores and hippocampal volumes. In a linear regression, AMT volume was a statistically significant predictor of patients' delayed visual memory scores (*β* = .8, *p* = .001) while neither the hippocampal volumes, the memory assessment version (WMS-III and CMS), nor the age at scan contributed significantly to the model (model's R^2^ adj. = .60, *p* = .002). In the controls, there were no correlations between memory measures and volumes of either the hippocampus or the thalamus.

## 4 Discussion

In a recent study, we reported a causal sequence from exposure to neonatal hypoxia-ischaemia leading to significant hippocampal pathology, in turn resulting in a pronounced deficit in episodic recall in the absence of deficits in semantic memory, working memory, academic attainments and intelligence. In patients with amnesia resulting from hypoxic-ischaemic events, the hippocampus is the structure predicted to exhibit the largest degree of atrophy. Here, however, we show that damage to the diencephalon also occurs in patients with hypoxia-induced DA. In addition to the atrophy of the hippocampus (Cooper et al., 2015, Isaacs et al., 2003), the mammillary bodies were damaged to such an extent that, in two thirds of the cases, they were not identifiable on conventional MRI images with 1 mm resolution (see Fig. 4). The DA patients also had a moderate degree of volume reduction in both the anterior and posterior divisions of the thalamus (see Fig. 6). The volume of the anterior-to-mid thalamic segment, AMT, which covers the anterior two-thirds of the total length of the thalamus, was highly correlated with the patients' visual memory performance. Each of these findings will be discussed in turn in the sections that follow.

### 4.1 Aetiology of the diencephalic damage

Given the large extent of the patients' hippocampal atrophy, the question arises as to whether the diencephalic damage is perhaps a secondary, anterograde effect of hippocampal degeneration. Due to their interconnections (for a review, see Aggleton et al., 2010), atrophy of the anterior thalamic nuclei and the mammillary bodies may occur as a distal effect of the hippocampal pathology. However, hippocampal damage is not expected to produce substantial volume reductions of the posterior nuclei of the thalamus. Since the AMT and the PT subdivisions were damaged to the same extent in the DA patients, it is likely that the thalamic volume reduction is at least in part an additional primary effect of the hypoxia-ischaemia. Indeed, in neonates, acute hypoxic-ischaemic events of high severity are known to produce significant thalamic injury (Chao et al., 2006, Latchaw and Truwit, 1995). However, the images available in the current study were not of sufficient resolution to permit segmentation of specific thalamic nuclei.

Although reports of hypoxia-ischaemia causing severe, and selective damage to the mammillary bodies are rare, extensive atrophy of this structure has been described in adults after prolonged heart failure (Kumar et al., 2009, Pan et al., 2013). Volume reduction in the medial nucleus of the mammillary bodies has also been reported following adult-onset damage to the hippocampus and fornix (Loftus et al., 2000, Schubert and Friede, 1979). Typically, anterograde degeneration of the mammillary bodies does not involve neuronal loss. The frequently occurring, and extensive loss or reduced volume of mammillary bodies in patients with DA could therefore suggest other causative factors. For example, Bleier (1969) described total loss of mammillary body neurons in newborn rabbits following removal of limbic cortex, through retrograde trans-synaptic degeneration. Importantly, the severity of this secondary damage to the mammillary bodies decreased with increasing age at injury, resulting in only a mild loss of neurons following injury in adulthood. It is therefore possible that the extensive atrophy observed in the DA patients is due to the early age of their injury. Indeed, in the current study, the mammillary bodies were still visible in the MRI of all three patients who had sustained injury during childhood (between 4 and 15 years of age), whereas in the cases with the most severe mammillary body damage, hypoxia-ischaemia had invariably occurred during the perinatal period.

Recently, Rosenbaum et al. (2014), using high resolution imaging, noted an absence of the mammillary bodies in one of the DA patients included in the current study (case HC in Rosenbaum et al., 2014; also referred to as case DA-6 in Adlam et al., 2005, 2009). The former authors speculated that this pathology resulted from a congenital abnormality rather than from a hypoxic-ischaemic injury associated with the patients' history of early-life respiratory dysfunction. In the current study we have identified a large number of cases with DA who had a documented hypoxic-ischaemic event, and whose mammillary bodies appear to be absent on their MRI scans. Given the retrospective nature of our study, we can only speculate on the mechanisms of injury in individual cases; nevertheless, it is now clear that an extreme loss of mammillary body volume can follow perinatal episodes of hypoxia-ischaemia.

### 4.2 Effects on memory function

We showed that patients with DA had deficits in recall of episodic memory, despite relatively preserved intelligence, semantic memory and recognition. Whereas we failed to find a correlation between hippocampal volumes and the patients' memory performance in the current study, we had previously shown that such a relationship exists in a larger sample of patients with a history of neonatal hypoxia-ischaemia, whose hippocampal volumes ranged from severely reduced to normal (Cooper et al., 2015; see also; Patai et al., 2015). These results are not necessarily inconsistent. It could turn out, that once a threshold of hippocampal atrophy is reached, the hippocampus is rendered non-functional (Squire & Wixted, 2011).

The mammillary bodies, the anterior and mediodorsal thalamic nuclei, and the mammillothalamic tract each have an established role in memory (Aggleton et al., 2010, Harding et al., 2000, Kopelman, 2002). In patients with a history of colloid cyst in the third ventricle, damage to the fornix and mammillary bodies contributed to their deficits in recall and recollection memory, but had no effect on their familiarity-based recognition memory (Denby et al., 2009, Tsivilis et al., 2008, Vann et al., 2009). On the other hand, patients with Korsakoff's syndrome (which is associated with more widespread damage to the diencephalon, frequently including the mammillary bodies as well as the anterior and mediodorsal thalamic nuclei) typically have deficits in both recall *and* recognition (Gold and Squire, 2006, Kopelman, 2002). However, impairments in recognition have also been associated with relatively selective damage to the anterior thalamic nuclei and the mammillary bodies (Cipolotti et al., 2008, Gold and Squire, 2006).

Mammillary bodies have been reported to contribute to spatial memory independent of their hippocampal inputs. Vann (2010) has shown that the mammillary bodies do not simply serve as a relay between the hippocampus and upstream structures. The medial mammillary body nucleus has unique contributions to processing of spatial information through its inputs from ventral tegmental area of Gudden. Therefore, the mammillary body damage reported here could have an additive detrimental effect on spatial memory function in patients with DA. However, examination of spatial memory scores obtained from the Boundary and Landmark Test (Guderian et al., 2015) in DA patients with absent versus those with visible MB did not reveal a significant impairment in the former subgroup. Furthermore, the extent of hippocampal damage is greater in those with absent MB compared to those with visible MB. Based on the available evidence, therefore, it is difficult to tease apart the additive effects of MB in our patients.

Finally, we found a relationship between the patients' thalamic volumes and their memory performance. Thus, those with intact volumes of AMT performed well on tests of delayed visual memory. This relationship could be attributed to a process of compensation associated with early pathology, although the mechanism of this is not yet known. Alternatively, certain cognitive components of the delayed visual memory tasks (e.g., visual processing, visual recognition, face perception) could be supported by the thalamic structures independent of the hippocampal network.

Importantly, our findings raise the possibility that the memory deficits in recall associated with DA are not solely a consequence of hippocampal damage. Rather, the DA patients' injury extends into the diencephalic network, and thus earlier conclusions regarding the source of their episodic memory impairment need to be reconsidered.

## Funding

This work was supported by the Medical Research Council (programme grant numbers G03000117/65439 and G1002276-E01/1), and the Intramural Research Program of the National Institute of Mental Health, National Institutes of Health, and supported by the National Institute for Health Research Biomedical Research Centre at Great Ormond Street Hospital for Children NHS Foundation Trust and University College London.

## Figures and Tables

**Table 1:** Results of neuroradiological examination. No abnormalities were detected in the parahippocampal gyrus (perirhinal, entorhinal, and parahippocampal cortices), thalamus, or the basal ganglia. Abbreviations: abn – abnormalities, bil – bilateral, CC – splenium of the corpus callosum smaller in size than the genu, in contrast to the reverse pattern seen in the healthy population (Yes/No), Cer – cerebellum, dil – dilated, Fx – fornix, Hipp – hippocampus, Lat v – lateral ventricle, MB – mammillary bodies, N – normal, PV WM – periventricular white matter, PVL – periventricular leukomalacia, Sm – small, V Sm – very small.

Patient	Hipp	Fx	MB	Cer	CC	PV WM	Lat v	Other
DA-1	Sm	Sm	Sm	Sm	No	Mild PVL	N	
DA-2	Sm	V Sm	Sm	Sm	Yes	PVL, Focal abn bil	Dil	
DA-5	Sm	V Sm	Sm	N	No	N	N	
DA-6	Sm	V Sm	Sm	N	Yes	N	Mild dil	
DA-9	Sm	Sm	N	N	Yes	N	Dil	
DA-10	Sm	N	N	N	No	N	N	
DA-12	Sm	Sm	N	N	Yes	Focal abn bil	Dil	
DA-13	Sm	Sm	Sm	Sm	No	Focal abn bil	N	
DA-14	Sm	Sm	Sm	N	No	N	N	
DA-15	Sm	V Sm	Sm	N	No	N	N	
DA-16	Sm	V Sm	Sm	N	Yes	N	N	a
DA-17	Sm	Sm	Sm	N	No	Focal abn R	N	
DA-18	Sm	Sm	Sm	N	No	N	N	
DA-19	Sm	Sm	N	N	No	Focal abn bil	V Mild dil	
DA-20	Sm	Sm	Sm	N	No	Focal abn bil	Mild dil	
DA-21	Sm	Sm	N	N	Yes	N	Mild dil	
DA-22	Sm	Sm	Sm	N	Yes	Focal abn bil, Diffuse abn	N	
DA-23	Sm	Sm	Sm	N	No	Focal abn bil	N	

a Focal abn Left Claustrum.

**Table 2:** Aetiology.

Patient	Aetiology	Age at injury
DA-1^a^	Prematurity, severe apnoea	Perinatal
DA-2	Cardiac arrest associated with maternal diabetes	Perinatal
DA-5	Foetal distress, respiratory problems	Perinatal
DA-6^b^	Prematurity, respiratory problems	Perinatal
DA-9	Epilepsy	4 years
DA-10	Theophylline toxicity leading to cardiac arrest	9 years
DA-12	Hypoglycaemia	15 years
DA-13	Prematurity, respiratory problems	Perinatal
DA-14	Respiratory problems following treatment for transposition of the great arteries	Perinatal
DA-15	Foetal distress, respiratory problems	Perinatal
DA-16	Foetal distress complicated by maternal pre-eclampsia	Perinatal
DA-17	Complications following treatment for transposition of the great arteries	Perinatal
DA-18	Second of twin birth, cardio-respiratory problems	Perinatal
DA-19	Acute hypoxaemic respiratory failure, Meconium aspiration syndrome	Perinatal
DA-20	Acute hypoxaemic respiratory failure, Prematurity	Perinatal
DA-21	Acute hypoxaemic respiratory failure, Prematurity, Persistent pulmonary hypertension	Perinatal
DA-22	Acute hypoxaemic respiratory failure, Prematurity, Sepsis	Perinatal
DA-23	Prematurity, Cardiac arrest	Perinatal

a Also referred to as Jon (e.g., Vargha-Khadem et al., 1997).

b Patient HC (Hurley, Maguire, & Vargha-Khadem, 2011; Rosenbaum et al., 2011).
